# Reducing environmental exposure to PPPs in super-high density olive orchards using UAV sprayers

**DOI:** 10.3389/fpls.2023.1272372

**Published:** 2024-01-04

**Authors:** Luis Sánchez-Fernández, María Barrera-Báez, Jorge Martínez-Guanter, Manuel Pérez-Ruiz

**Affiliations:** ^1^ Departamento de Ingeniería Aeroespacial y Mecánica de Fluidos, Área de Ingeniería Agroforestal, Universidad de Sevilla, Seville, Spain; ^2^ Digital Marketing Manager Iberia at Corteva Agriscience, Sevilla, Spain

**Keywords:** plant protection product application, autonomous UAV, spray drift, olive, precision farming

## Abstract

The increasing demand for optimizing the use of agricultural resources will require the adoption of cutting-edge technologies and precision farming management. Unmanned Aerial Vehicle (UAV) sprayers seem promising due to their potential to perform precision or spot spraying, particularly in woody crop environments where total surface spraying is unnecessary. However, incorporating this technology is limited by the lack of scientific knowledge about the environmental risks associated with UAV sprayers and the strict legal framework. Nonetheless, these spraying systems’ characteristic downwash airflow and the limited swath width can potentially mitigate drift in hedgerow crops. During our study we performed comparative studies aimed to compare the airborne drift, soil, and crop depositions between a conventional orchard sprayer and a UAV sprayer in a commercial superhigh-density orchard in the South Iberian Peninsula in 2022. Our findings reveal that, in superhigh-density olive orchards, the UAV sprayer presents a substantial reduction in airborne drift, while soil depositions showed no significant differences compared to those of a conventional terrestrial orchard sprayer. Crop depositions were significantly lower when utilizing the UAV sprayer. These results suggest that introducing UAV spraying technology in Mediterranean agricultural systems, under specific scenarios, can effectively reduce the environmental impact of crop spraying and encourage the responsible use of plant protection products (PPPs).

## 1 Introduction

The United Nations expects the population to grow by two billion people over the next 30 years (United Nations, 2022), for which agriculture must provide food, fiber, and fuel. The estimated increase in calorie consumption that comes with the economic growth of developing countries will require an expansion of approximately 70% in agricultural production (Searchinger et al., 2018). Increasing agricultural yield is not an easy task, especially in the context of climate change, which is expected to cause a significant reduction in precipitation in the Mediterranean region, one of the main agricultural areas in the world (Masson-Delmotte et al., 2019). Increasing the agricultural area at the expense of natural ecosystems is not sustainable, so the increased agricultural yield must come from sustainable intensification. Moreover, there is increasing concern about the environmental impact of PPPs. This concern has resulted in strategies, such as the Farm to Fork initiative, that aims to reduce the use of PPPs in Europe by 50% over the next decade.

Some technologies with the potential to contribute to solving this challenge have already been developed. Unmanned aerial vehicles (UAVs) are becoming increasingly popular in the agricultural industry due to their adaptability and versatility. UAVs are now being utilized for various agricultural tasks, including the precise application of PPPs with a high spatial resolution (Huang et al., 2009). In some situations, spraying UAVs might be more suitable than conventional spraying systems since they can spray areas that are difficult for workers or machinery to access, such as hilly or muddy plots. Some studies suggest that the use of spraying UAVs has several advantages over conventional terrestrial spraying systems, especially when compared to orchard (Sánchez-Fernández et al., 2023) and backpack sprayers (Wang et al., 2018; Sarri et al., 2019; Xiao et al., 2020).

Spraying UAVs can perform variable and spot spraying, reducing the application’s environmental impact and contributing to the sustainability of agricultural systems. They are more time efficient than conventional terrestrial sprayers, and they can spray approximately 4ha·h^-1^, a significantly higher surface than a knapsack sprayer (Giles and Billing, 2015), while reducing the exposition to the operator. Furthermore, several studies suggest that the downwash airflow generated by the UAVs’ rotors may contribute to the penetration of the spray into the crop (He et al., 2017; Tang et al., 2017). Moreover, batteries power most commercial spraying UAVs, and their use might contribute to reducing the dependency on fossil fuels. However, there are still unknown aspects of the flight and structural parameters of UAVs that might affect the spray depositions, such as the distance between the rotors, their number, or the lifted weight (da Cunha et al., 2021). More studies in this field are needed to fully understand how all these parameters affect spraying UAVs’ depositions.

Spraying UAV technology, while promising, still faces significant limitations. The limited payload capacity of these systems restricts their application to ultra-low volume rates. Technologically, the payload limitation is being overcome by developing larger and heavier UAVs. However, one of the most vital limitations of this technology, especially in Europe, is the strict legal framework. UAV spraying is considered aerial spraying in most parts of Europe and, consequently, banned, except under exceptional circumstances and with minimal active ingredients approved. Comparative studies such as this are needed to determine if, under specific scenarios, UAV spraying systems can help reduce environmental exposure to PPPs compared to conventional terrestrial orchard sprayers.

Olive tree (*Olea europaea*) is the main permanent crop worldwide and one of the main crops in the Mediterranean region, covering 11.5 million hectares. Olive production is restricted to the Mediterranean climate areas, but a globally dispersed growing demand exists. As a result, the surface dedicated to olive orchards increases steadily to 162,000 hectares yearly (Vilar et al., 2018). Recently, superhigh-density orchards have gained prominence and represent most new plantations. Moreover, some traditional olive orchards are converted to superhigh-density or hedgerow orchards yearly due to their reduced human labor requirements, earlier returns on investment, consistency in yield, and efficient management (Lindell et al., 2023). Furthermore, some studies suggest that super high-density orchards may have some environmental benefits over traditional production systems, and they exhibit a lower impact on climate change per ton of production (Ben Abdallah et al., 2021). Currently, the spraying of PPPs in super high-density olive orchards is performed by terrestrial mist blowers. Introducing spraying UAVs in olive orchards has the potential to mitigate the environmental impact of these operations under specific conditions, supporting the sustainability of agricultural systems. Some studies assessing the drift generated by UAV sprayers have been published (Liu et al., 2020; Wang et al., 2021; Dengeru et al., 2022; Li et al., 2022). Nevertheless, the deposition parameters and airborne drift associated with UAV sprayers in superhigh-density olive orchards are still unknown. Our study is the first to compare the airborne drift, soil, and crop depositions generated by a UAV sprayer to those caused by a conventional orchard sprayer in super-high density olive orchards.

This study assesses the potential advantages of UAV spraying systems compared to conventional terrestrial systems concerning airborne drift and crop and soil depositions within specific conditions, particularly in superhigh-density hedgerow olive orchards. This study involves a comparative analysis of the airborne drift, crop, and soil depositions resulting from using a UAV sprayer and conventional terrestrial mist blower. Our trials were conducted in a representative commercial superhigh-density olive orchard whose characteristics and agricultural practices are common to orchards of the same type in the Mediterranean region. Our hypothesis states that UAV sprayers may generate less airborne drift while maintaining similar soil depositions to conventional terrestrial atomizers. Conventional orchard sprayers typically project the spray horizontally onto the crop’s canopy, resulting in a substantial portion of the applied volume passing through the canopy as drift.

Conversely, UAVs spray vertically, directing the spray downward over the crop with the assistance of the downwash airflow generated by the rotors. This downward airflow might promote spray penetration into the canopy, mitigating airborne drift. In specific scenarios, adopting UAV sprayers can help reduce the use of PPPs, consequently safeguarding and expanding areas of environmental interest adjacent to agricultural areas.

The chosen crop for our study is superhigh density olive-orchards. These orchards hold significant importance in the region, accounting for 46.7% of the total agricultural surface (SIGPAC, 2021). Andalusia contains 61% of the total olive surface of Spain, the main olive producer in the world.

## 2 Materials and methods

### 2.1 Study area and target crop

The focus crop of this study was a superhigh-density olive (*Olea europaea*) orchard. Drift trials were conducted from June to September 2022, during phenological growth stages 71 and 75 according to the BBCH scale. The study occurred at Bujalmoro farm (37°13’N, 5°55’W), a commercial superhigh-density olive orchard in Andalusia (South Iberian Peninsula). The olive trees were fully developed and planted at a planting frame of 4 m x 1.5 m (1667 trees·ha^-1^) in 2018. The trees’ height measured from the ground was 3 m, the hedgerow width was 1.66 m, and the measured canopy volume was 10555 m^3^·ha^-1^. Andalusia has typical Mediterranean climatic conditions characterized by mild winters and hot and dry summers. The average rainfall and reference evapotranspiration (ET_o_) registered in the orchard have been 484 mm and 1442 mm, respectively, for the last 25 years. The characteristics and the management of the orchard are representative of the superhigh-density commercial orchards of the region and the Mediterranean area.

### 2.2 Spraying systems

#### 2.2.1 Orchard sprayer

**Figure 1:**
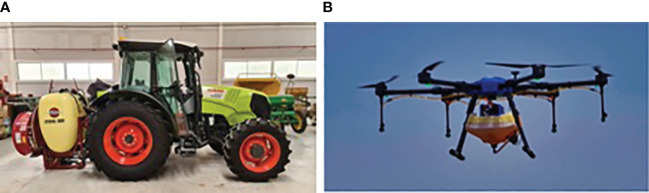
**(A)** Typical orchard sprayer currently used by farmers and **(B)** UAV sprayer used in our study.

In our study, we compared the airborne drift, soil, and crop depositions of two spraying systems: a conventional orchard sprayer and an autonomous UAV spraying system (**Figure 1**). A tractor-mounted mist blower (Zebra Axial 600, HARDI International, Nørre Alslev, DK) was attached to a Claas Elios 240 (Claas, Harsewinkel, DE), 73 kW tractor. This is the typical sprayer used in the region’s olive orchards and woody crops. The mist blower has six ceramic hollow cone nozzles (Albuz ATR-80, Solcera, Evreux, FR) on each side. The two bottom nozzles were yellow, the two middle ones were orange, and the top were red. The top red nozzle at each side of the mist blower was closed to adjust the sprayed area to the crop’s height. The mist blower operated with the rear intake at 280 rpm and 10 bar, spraying 13.5 L·min^-1^. The final application rate at a 0.7 m·s^-1^ speed was 800 L·ha^-1^. The proper function of the mist blower was checked following ISO 16122 (2015) under the same working conditions.

#### 2.2.2 Autonomous UAV sprayer

The UAV spraying system is a prototype hexacopter equipped with an RTK-GNSS system (Here3+, HexAero Pte. Ltd, SG), a 16 L tank, and four green hollow cone nozzles (KZ-80 06, Ningbo Licheng Agricultural Spray Technology Co., Ltd, Yuyao, CN) placed just below the frontal rotors. Determining the swath width is a crucial factor in UAV sprayers. We conducted an indoor trial using three sampling lines to evaluate the spraying UAV swath width without the influence of the wind. In each sampling line, we fixed 26 x 76 mm water-sensitive papers (Syngenta, Basel, CH) every 0.4 m; each line was 3 m from each other. During the swath width trials, we assessed the depositions of the UAV sprayer at 1 m, 2 m, and 3 m high. Flight speed was the same used during the field trials, 1.5 m·s^-1^. The spraying height of the field trials was 1.5 m above the canopy. With these flight parameters, the final application rate was 40 L·ha^-1^. The image analysis software ImageJ (ImageJ 1.52p, NIH, EEUU) analyzed the water-sensitive paper. The proper function of the nozzles in the UAV sprayer was checked following ISO 16122 (2015).

### 2.3 Experimental design

**Figure 2:**
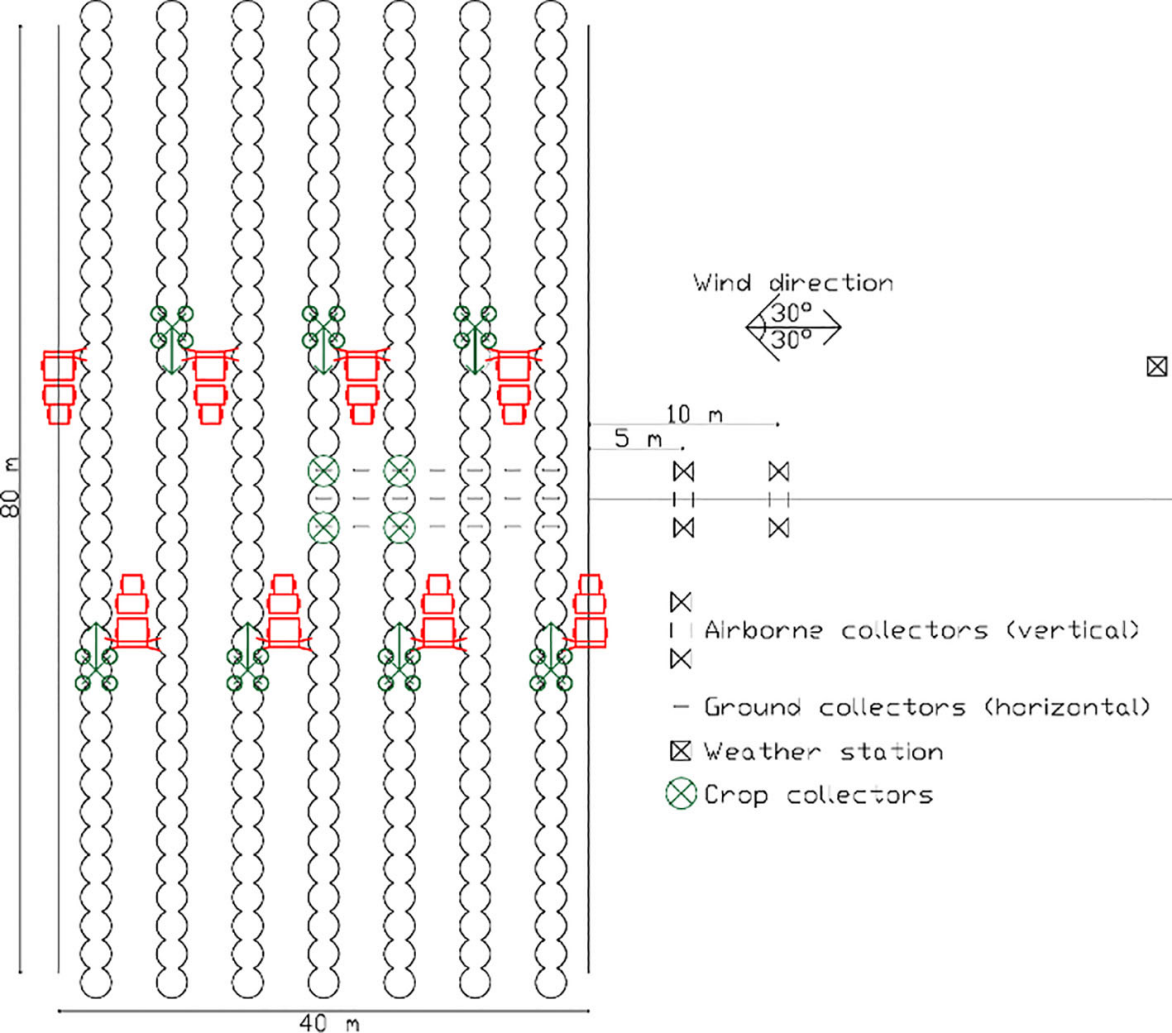
The layout of the experimental site. Airborne spray drift collectors were placed in the drift area, while soil and crop collectors were placed in the spraying area. Weather stations were placed in the drift area.

The experimental plot was surrounded by farmlands covered in grassy crops that were mowed and ploughed to establish a drift measurement area free of crops and obstacles of 40 m in length and 50 m wide, meeting the requirements of ISO 22522 (2007) and ISO 22866 (2005), in which the trials were conducted. The spraying area measured 80 m in length and 40 m in width. Soil and crop deposition trials were performed together in the spraying area, while airborne drift trials occurred in the adjacent drift area. The area was sprayed three times for each trial and sprayer. After each repetition, collectors (**Figure 2**) were meticulously collected and replaced to ensure accurate data collection and analysis.

Weather conditions were monitored using three weather stations (WH3000SE PRO, Shenzhen Fine Offset Electronics Co., Ltd, Guangdong, CN) placed 1 m above the canopy in the drift area. Only trials carried out under acceptable weather conditions according to ISO 22866 (2005) were considered for this study. As for the tracer, we employed Tartrazine (E-102, Seimex & Procona Ltd., Valencia, ES). Given the differences in the spraying parameters between the two tested spraying systems (conventional vs ultra-low volume spraying), we applied different concentrations of tracer to ensure the same amount of tracer was sprayed in the spraying area. The conventional terrestrial sprayer utilized a 0.6 g·L^-1^ concentration, while the UAV sprayer sprayed a higher 12 g·L^-1^ concentration. Following application, we allowed the collectors to dry for five minutes before carefully placing them into individual zip bags. These bags were then stored in cool and dark conditions to prevent degradation. Samples were taken from the tanks of both spraying systems for a thorough analysis to determine the precise amount of tracer sprayed.

**Figure 3:**
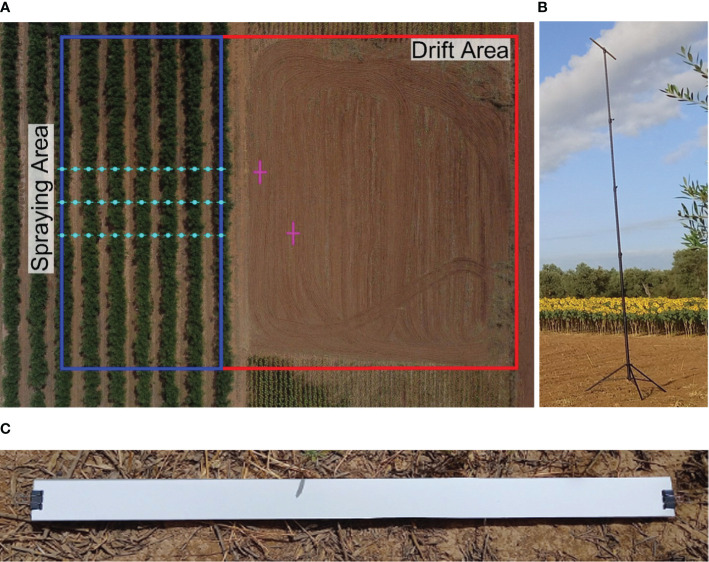
**(A)** Aerial view of the experimental area showing the position of soil and airborne collectors. **(B)** Array used to place the airborne collectors. **(C)** The soil collector was used in our trials.

The airborne drift was measured 5 and 10 m downwind from the sprayed area. The collector used was a PETG filament with a diameter of 2.85 mm (KIMYA, Nantes, FR), which was vertically arranged on 6 m tall poles in an array. We placed two filaments at each sampling distance. Sampling of airborne drift was performed at regular intervals of 0.5 m, starting from the ground level up to a height of 6 m. To evaluate soil depositions under the hedgerow and interrow areas, we established three sampling lines perpendicular to the hedgerow, spaced every 5 m at the center of the spraying area (**Figure 3**). Each sampling line was comprised of a total of 7 data collection points. This setup allowed us to sample intra-row and interrow surfaces within the spraying area. To measure crop depositions, we chose four representative trees around the spraying area’s center (**Figure 2**). To understand the spray distribution within the canopy, each selected tree was sampled at three different heights: 1, 2, and 3 m from the ground. We evenly positioned four absorbent paper collectors (CANSON, Annonay, FR) at each height per sampling tree, each collector had a surface of 5 x 5 cm.

### 2.4 Depositions calculation

Once in the laboratory, the tartrazine from each collector was extracted using a known volume of deionized water. To determine the amount of tartrazine present, the absorbance of the wash-off water was measured at a wavelength of 425 nm using a spectrophotometer (Cary UV-Vis Compact, Agilent Technologies, Inc., Santa Clara, USA) **Equation 1**. Blank collectors were analyzed to eliminate any potential influence of the collectors and deionized water on the absorbance readings. Using a calibration curve previously done with the same spectrophotometer, the spray deposit of each collector was calculated based on the amount of tartrazine extracted **Equation 2**. Additionally, we analyzed the samples extracted from the sprayers’ tanks before and after each repetition to determine the precise concentration of tartrazine sprayed:


(1) $$SD=\frac{\left(\rho_{smpl}-\rho_{blk}\right)\cdot F_{cal}\cdot V_{dil}}{\rho_{spray}\cdot A_{col}}$$

where *SD* represents the deposit extracted from each collector (µL·cm^-2^); *ρ*_smpl_, the absorbance (dimensionless) of the sample washing; *ρ*_blk_, the absorbance (dimensionless) of the blank collectors washing; *F*_cal_, the calibration factor; *V*_dil_, the volume of the deionized water used to dilute the tracer from the collector (µL); *ρ*_spray_, the absorbance (dimensionless) of the tank solution; and *A*_col_, the area of the collector (cm^2^).

The percentage of spray drift on a collector (*D*_%_) was calculated considering the projected area of each collector. Finally, the deposit was expressed as a percentage of the total volume sprayed in the same area using the following expression:


(2) $$D_{\%}=\frac{SD\cdot 10000}{\beta_{v}}$$

where βv is the spray application volume per hectare (L·ha^-1^) and is given by the following **Equation 3**:


(3) $$\beta_{v}=\frac{T_{flow}\cdot 600}{R_{spac}\cdot V}$$

where *T*_flow_ is the total nozzle flow rate (L·min-1); *R*_spac_ is the distance between crop lines (m); and *V* is the velocity of the sprayer (km·h^-1^).

### 2.5 Data analysis

The effect of the spraying system on the airborne, soil, and crop depositions at every distance and height was evaluated using a one-way analysis of variance (ANOVA) coupled with Fisher’s least significant difference (LSD) test (Fisher, 1936). Before conducting the analysis, we ensured that the data met the necessary assumptions for these tests. The Shapiro-Wilk test (Shapiro and Wilk, 1965) was employed to assess the normality of the data, while Levene’s test (Levene, 1960) was used to examine the homogeneity of variance. All statistical tests were carried out with a confidence level of 95%. The results were analyzed using the R statistics software (R Core Team, 2022).

## 3 Results

### 3.1 Sprayer UAV swath width

**Figure 4:**
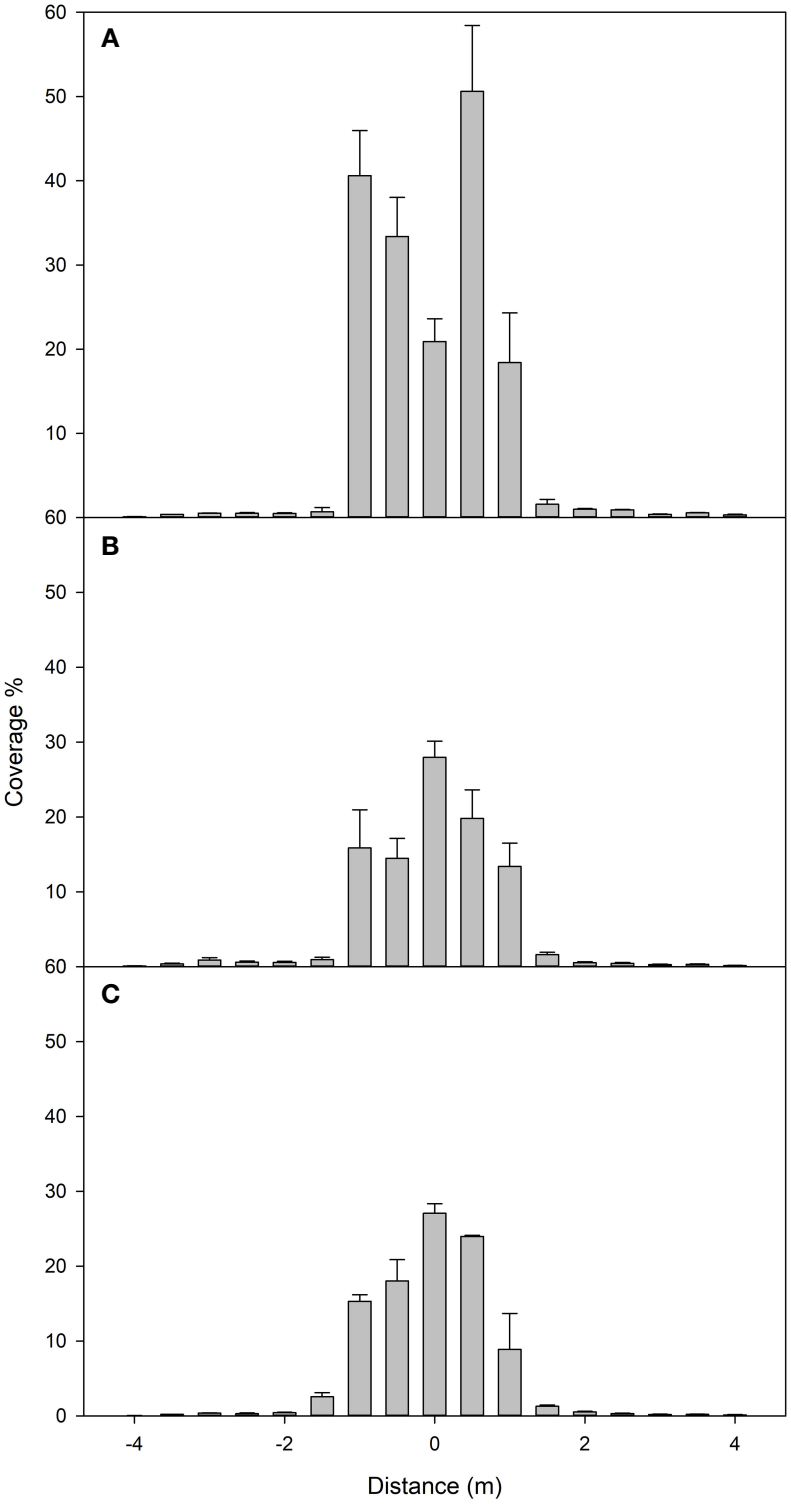
Coverage % measured with water-sensitive paper flying at 1 m **(A)**, 2 m **(B)**, and 3 m **(C)** above the sampling lines. Vertical bars show the standard error (SE). Distance 0 shows the flight path of the UAV sprayer.

**Figure 4** presents spray coverage distribution at different horizontal distances from the flight path of our UAV, this swath width trial was repeated at three different heights: 1 m (a), 2 m (b), and 3 m (c). As shown in **Figure 4**, almost no coverage is detected after 1.5 m from the UAV’s flight path for the three tested heights. As **Figure 4** suggests, we observed a higher spray coverage when flying lower. Still, after 2 m high, the coverage seems to stabilize with height, and no further reduction in coverage is observed when increasing height to 3 m. When analyzing the standard error across different height measurements, a consistent decrease is observed with increasing heights, indicating a trend toward spray homogenization at increasing heights. Our data (**Table 1**) also shows how the variation coefficient decreases with height.

**Table 1:** Results of the swath width trials performed indoors.

Flying Height (m)	Swath width (m)	CV (%)
1	2.25	28.13
2	2.5	27.13
3	2	25.84

### 3.2 Airborne spray drift

**Figure 5:**
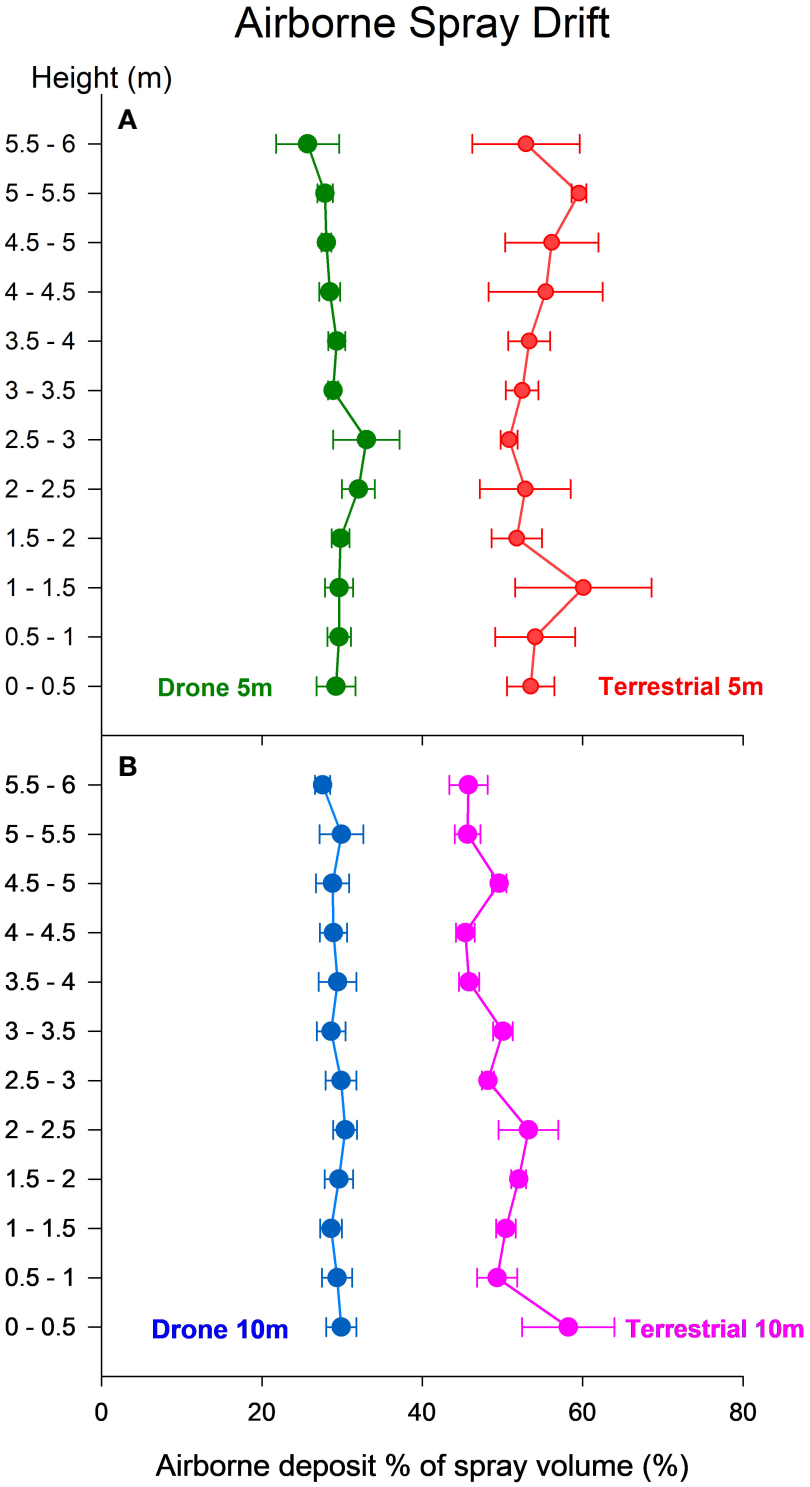
Airborne deposit (% of spray volume) measured to a height of 6 m above the ground: Aerial depositions measured 5 m downwind from the spraying area **(A)**; Aerial depositions measured 10 m downwind from the spraying area **(B)**. Each point represents the average of three spraying events, and horizontal bars show the standard error (SE).

Values of airborne spray drift show significant differences between both sprayers at 5 and 10 m downwind from the sprayed area for every height (p< 0.05). As shown in **Figure 5**, the statistical analysis did not show significant differences in the airborne deposit generated by the UAV sprayer at 5 and 10 m downwind from the sprayed area. However, the terrestrial sprayer caused significantly less airborne drift at 10 m downwind from the sprayed area when compared to the airborne drift captured by the sampling array placed at 5 m. Our results show that the UAV sprayer generated significantly less airborne drift under our conditions than our conventional terrestrial orchard sprayer.

### 3.3 Soil depositions

**Figure 6:**
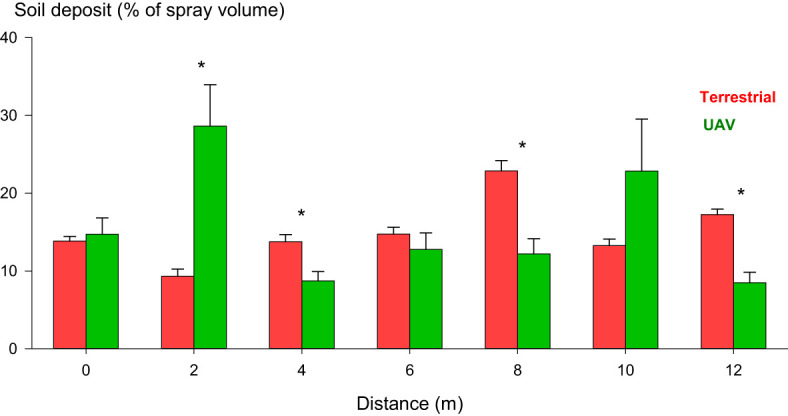
Soil depositions (% of spray volume) were collected intra-row (0, 4, 8, and 12 m) and interrow (2, 6, 10 m). Distances are measured upwind from the border of the spraying area. Vertical error bars represent the standard error (SE). Positions with statistical differences between the sprayers are marked with *.

Our soil deposition results (**Figure 6**) suggest that the total % of spray volume that reached the soil was similar between the sprayers, with no statistically significant differences in the total % of spray volume collected. However, our results showed statistically significant differences between the sprayers at specific positions. At intra-row places (4, 8, and 12 m), the terrestrial orchard sprayer demonstrated significantly higher soil depositions than the UAV sprayer. The UAV sprayer exhibited more variability in soil depositions at each sampled position when compared to the terrestrial orchard sprayer.

### 3.4 Crop depositions

**Figure 7:**
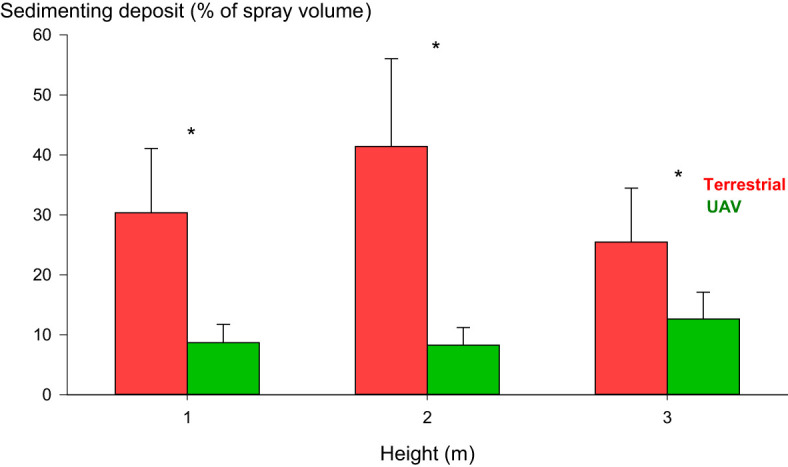
Crop depositions (% of spray volume) collected at 1, 2, and 3 m height measured from the ground. Vertical error bars show the standard error (SE). Positions with statistical differences between the sprayers are marked with *.

As some of our data did not meet the assumptions for ANOVA, we also employed the Kruskal-Wallis test (Kruskal and Wallis, 1952). As depicted in **Figure 7**, our analysis revealed distinctions in crop deposition between the terrestrial orchard sprayer and the UAV sprayer at each sampled height. Specifically, the terrestrial orchard sprayer showed a significantly higher crop deposition at every sampled height than the UAV sprayer. However, the data dispersion suggests that the terrestrial orchard sprayer displayed less homogeneity in crop depositions at every sampled height. Moreover, the spatial distribution of crop deposition varied between the two sprayers. The terrestrial orchard sprayer generated higher depositions at 2 m above the ground in the middle section of the hedgerow. By contrast, the UAV sprayer showed a higher crop deposition at the top part of the canopy, at a height of 3 m above the ground. Our data did not show significant differences between the depositions generated by the UAV sprayer at 1 and 2 m from the ground.

## 4 Discussion

We evaluated airborne drift and mass balance distribution to assess spray deposition and drift. In our analysis of swath width, we observed a decrease in the coefficient of variation (CV) as the height increased. This might suggest that the spray’s distribution trended to become more homogenized at higher altitudes, potentially due to the effect of the turbulence generated by the downwash airflow. In our study, the flight height had a limited influence on the UAV’s swath width. This suggests that, within the range of 1 to 3 m, the swath width was more dependent on other factors, such as the spacing between the rotors and the characteristics of the downwash airflow generated by these rotors.

Our results suggest that UAV sprayers generate significantly less airborne drift than conventional orchard sprayers. These results align with the findings of previous work (Sánchez-Fernández et al., 2023), which observed reduced sedimented spray drift from a UAV sprayer compared to a conventional terrestrial orchard sprayer. Our experiments observed no statistically significant differences in the airborne spray drift among the various heights tested for any of the studied sprayers. However, our data shows a trend of increasing airborne spray drift as height decreases; this phenomenon seemed stronger for the terrestrial orchard sprayer at 10 m downwind from the spraying area. This trend is consistent with observations in similar studies (van de Zande et al., 2014; Torrent et al., 2017; Gil et al., 2018; Wang et al., 2021).

The influence of the vortex generated by rotor blades interacting with the air and the interplay between the UAV’s wind field and the canopy can affect droplet deposition, as suggested in previous studies (Xue et al., 2014; Guo et al., 2019). Spraying at lower altitudes facilitates a more efficient projection of the spray onto the crop below, whereas, at higher altitudes, the vortex can emerge, leading to increased drift. Our airborne spray drift results support our hypothesis that downward spraying of the UAV sprayer results in less drift than a terrestrial orchard sprayer, even at heights of 1.5 m above the olive hedgerow, corresponding to a spraying height of 4 m above the ground. The mentioned vortex occurs at this height and induces significantly more drift than lower-altitude spraying. Despite the presence of the vortex and its potentially harmful effects, the UAV sprayer still generated significantly lower airborne spray drift than the terrestrial orchard sprayer.

In terms of soil depositions, our study revealed significantly higher depositions for the terrestrial orchard sprayer in the intra-row sampled positions. However, no statistically significant differences were observed in soil depositions for the inter-row sampled positions. Additionally, our results suggest a higher spray variability when using the UAV sprayer than the conventional orchard sprayer. These soil spray deposition results align with previous studies, suggesting that UAV sprayers tend to have lower droplet density and deposition uniformity. The spray drift generated by the conventional orchard sprayer while spraying adjacent rows generated soil depositions comparable to those generated by the UAV sprayer. Although no statistically significant differences were observed in the soil depositions collected from the inter-row positions, this result holds significant meaning, especially considering that the terrestrial orchard sprayer was a mist blower whose spraying area was adapted to the canopy’s surface, that it did not spray the soil, and that the swath width of our UAV sprayer was wider than the hedgerow width. In hedgerow crops, the swath width of the UAV sprayer becomes especially important, ideally it should be equal to or narrower than the hedgerow width to prevent the spraying of the crop’s interrow space. In our study, the swath width of the UAV sprayer was 2.25 m, while the hedgerow width was, on average, 1.66 m. This excessive swath width may be why we did not find significant differences in soil depositions between the two tested sprayers. Further optimization of this parameter may reduce the UAV’s soil depositions. Maintaining an appropriate swath width is essential to prevent off-target spray drift, which can decrease spraying efficiency and increase environmental exposure to plant protection products (PPPs). Commercial UAV sprayers cannot adapt their swath width to woody orchards’ heterogeneous and changing characteristics. Further research is needed in this aspect to ensure the precision of this technology in spraying applications.

Regarding crop depositions, our results show that the UAV sprayer’s crop depositions are significantly limited compared to the crop depositions generated by the conventional orchard sprayer. This behavior can be attributed to the distinct spraying parameters employed by the two spraying systems: the terrestrial orchard sprayer applies a high volume laterally to the canopy, while the UAV sprayer projects an ultra-low volume spray downward from the top of the canopy. The higher spray volume of the terrestrial orchard sprayer promotes better penetration but also leads to increased run-off from the canopy to the soil, which explains the higher soil depositions detected when using the conventional orchard sprayer. The terrestrial orchard sprayer generated more crop depositions in the middle section of the hedgerow, while the UAV sprayer produced more crop depositions at the top part of the canopy. This characteristic crop deposition pattern observed in the7nbsp;UAV sprayer is expected, given that drones spray from above the canopy. The phenomenon has been previously documented (Zhang et al., 2016; Martinez-Guanter et al., 2020; Li et al., 2021). The UAV sprayer did not exhibit statistical differences in crop depositions at the middle and lower levels of the canopy. This crop deposition pattern suggests that the downwash airflow associated with the UAV sprayer promotes better penetration and homogenizes the spray distribution at the middle and lower sections of the canopy.

## 5 Conclusion

This study demonstrates that UAV sprayers offer a significant advantage in reducing airborne drift compared to conventional orchard sprayers, particularly in super-high-density olive orchards. Our results also suggest that UAV sprayers generate soil depositions similar to conventional orchard sprayers. However, the practicality of using UAV sprayers may vary depending on the specific context and the desired crop depositions. Our findings suggest that UAV sprayers may be particularly well-suited for systemic or bait products that do not require extensive crop coverage for their efficacy. Moreover, in scenarios where spot spraying is necessary, UAV sprayers could prove valuable and efficient. Introducing UAV sprayers for systemic or bait PPPs, particularly in cases where ultra-low volume or spot spraying is effective, seems promising for reducing environmental exposure.

The findings presented in this work can be useful for quantifying the implications and the impact of the introduction of UAV sprayers in the Mediterranean agricultural environment and assessing the benefits of this technology to reduce the use and mitigate the consequences of plant protection product spraying. Introducing UAV sprayer technology might offer advantages in reducing the usage of PPPs and addressing their environmental impacts. However, further research is needed to fully understand how the spraying parameters affect the performance of UAV sprayers and to find ways of adapting UAV sprayers’ swath width to the requirements of each crop.

## Data availability statement

The raw data supporting the conclusions of this article will be made available by the authors, without undue reservation.

## Author contributions

LS-F: Data curation, Formal analysis, Investigation, Methodology, Writing – original draft, Writing – review & editing. MB-B: Data curation, Investigation. JM-G: Conceptualization, Funding acquisition. MP-R: Funding acquisition, Investigation, Writing – original draft.
