# Supplementary figures and images for: Reducing environmental exposure to PPPs in super-high density olive orchards using UAV sprayers
Source: Front Plant Sci. 2024 Jan 4;14:1272372. doi: 10.3389/fpls.2023.1272372 (PMC10794436; doi:10.3389/fpls.2023.1272372)

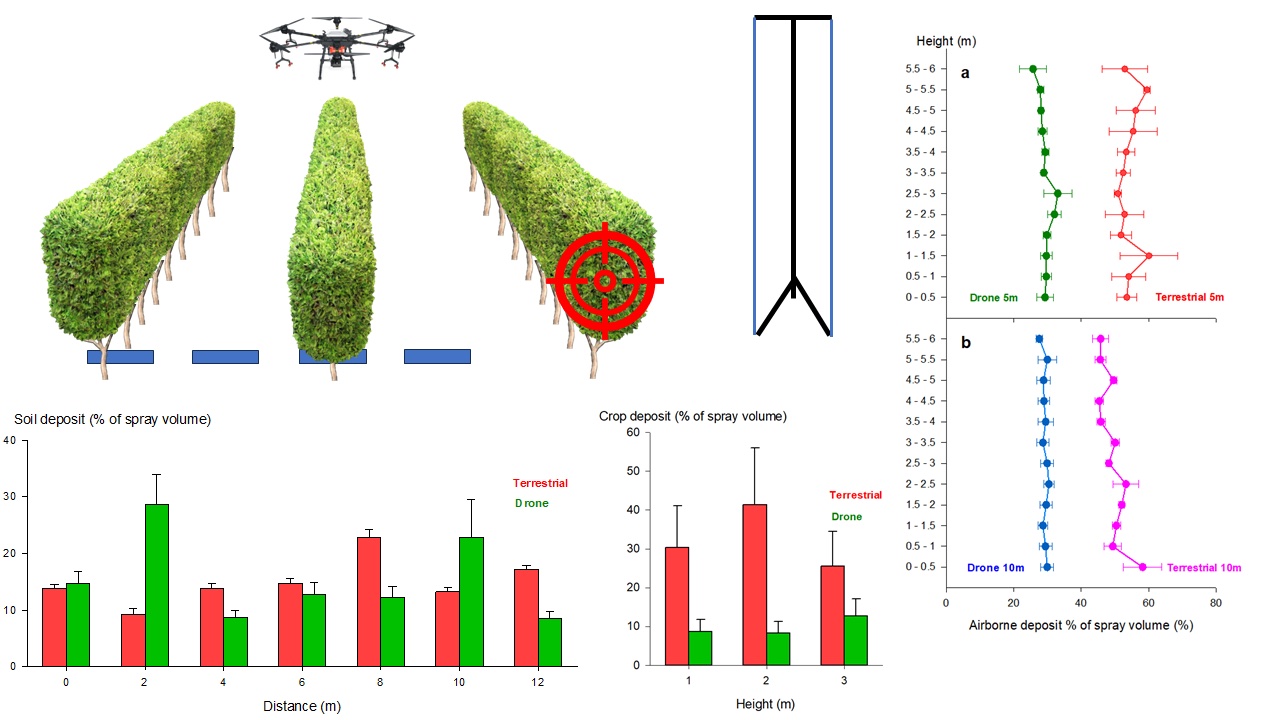

Supplement: Supplementary file 1 [file Image_1.jpeg]
